# A Review of Remote Anesthesia Supervision Models in Rural and Underserved Settings Using Teleanesthesiology Platforms

**DOI:** 10.51894/001c.164388

**Published:** 2026-08-01

**Authors:** Lauren Amoo, Justin Ma, Richa Patel, Maryam Khan, Jennifer Zora, Esha Elahi, Julia Vinagolu-Baur, Kelly Frasier

**Affiliations:** 1 College of Osteopathic Medicine, Michigan State University

## 42

### Introduction

Anesthesia workforce shortages severely limit surgical capacity and perioperative safety in rural and underserved regions, leading to delays, cancellations, and reduced access to time-sensitive procedures. Remote anesthesia supervision via teleanesthesiology platforms—using high-resolution audiovisual interfaces, real-time physiologic data streaming, and integrated electronic documentation—enables board-certified anesthesiologists to oversee on-site certified registered nurse anesthetists (CRNAs) or general practitioners in resource-limited settings. Pilot programs and clinical studies suggest comparable outcomes to in-person models for intraoperative complications, anesthetic depth control, and postoperative recovery, while facilitating subspecialty input for complex cases (e.g., high-risk obstetric, pediatric, or cardiovascular surgeries) without patient transfers.

### Objectives

This review aims to synthesize evidence on the efficacy, benefits, and barriers of teleanesthesiology for remote supervision and hypothesize that standardized implementation, regulatory alignment, and infrastructure improvements can establish it as a scalable, equitable extension of anesthetic practice to expand safe surgical care in structurally underserved communities.

### Methods

Narrative review of published literature, pilot programs, and clinical reports (2019–2025) on teleanesthesiology applications, including studies evaluating remote supervision outcomes, patient satisfaction, complication rates, and barriers (e.g., licensure, broadband, medico-legal issues). Sources include peer-reviewed journals and programmatic descriptions focusing on rural/underserved implementations.

### Results

Existing models demonstrate high patient satisfaction (93–95% in some reports) and improved decision-making for on-site providers (e.g., 87% of CRNAs in one survey). Comparable safety profiles to in-person care are reported in pilots, with no significant increases in complications. Benefits include expanded access and targeted subspecialty support. Key barriers persist: fragmented state licensure, inconsistent credentialing, limited broadband, and unresolved liability/shared responsibility frameworks, including concerns over accountability, consent, and escalation thresholds.

### Discussion

Teleanesthesiology offers a promising, high-acuity solution to anesthesia shortages, enabling expert remote oversight and advancing perioperative equity. However, full integration requires regulatory harmonization, standardized clinical protocols, infrastructure investment, and reconceptualization of practice models. With these addressed, remote supervision can legitimately extend anesthesiology’s reach, enhancing safety, access, and justice in underserved areas.

